# Candidate genes for cooperation and aggression in the social wasp *Polistes dominula*

**DOI:** 10.1007/s00359-018-1252-6

**Published:** 2018-02-27

**Authors:** Fabio Manfredini, Mark J. F. Brown, Amy L. Toth

**Affiliations:** 1 School of Biological Sciences, Royal Holloway University of London, Egham, UK; 2 Departments of Ecology, Evolution, and Organismal Biology and Entomology, Iowa State University, Ames, IA USA

**Keywords:** Wasp foundress, Dominance behaviour, Social aggression, Vitellogenin, Gene co-expression network.

## Abstract

Cooperation and aggression are ubiquitous in social groups, and the genetic mechanisms underlying these behaviours are of great interest for understanding how social group formation is regulated and how it evolves. In this study, we used a candidate gene approach to investigate the patterns of expression of key genes for cooperation and aggression in the brain of a primitively eusocial wasp, *Polistes dominula*, during colony founding, when multiple foundresses can join the same nest and establish subtle hierarchies of dominance. We used a comparative approach to select candidate genes for cooperation and aggression looking at two previously published studies on global gene expression in wasps and ants. We tested the expression of these genes in *P. dominula* wasps that were either displaying aggressive behaviour (dominant and single foundresses) or cooperation (subordinate foundresses and workers) towards nestmates. One gene in particular, the egg yolk protein *vitellogenin*, known for its reproductive role in insects, displayed patterns of expression that strongly matched wasp social rank. We characterize the genomic context of *vitellogenin* by building a head co-expression gene network for *P. dominula*, and we discuss a potential role for *vitellogenin* as a mediator of social interactions in wasps.

**Electronic supplementary material:**

The online version of this article (10.1007/s00359-018-1252-6) contains supplementary material, which is available to authorized users.

## Introduction

The evolution and mechanisms of cooperation are major areas of interest in animal behaviour, as cooperative behaviour is implicated in major transitions in evolution and is central to the success of our own species (Szathmáry and Maynard Smith 1995). The occurrence of social behaviours that give rise to cooperative groups and societies is often accompanied by changes in the occurrence of aggressive behaviour, with shifts toward reduced intragroup aggression and enhanced intergroup aggression in more highly social species. Nonetheless, in some animal societies, intragroup aggression is an important facet of group organization, providing group members with a social structure across which resources and reproductive opportunities may be divided (West 1967). There has been increasing interest in studying the mechanisms that regulate both cooperation and aggression in animal societies, and some studies suggest there may be conserved molecular mechanisms across a wide variety of social species, from bees to birds to mammals (Rittschof et al. 2014).

One of the best animal groups for dissecting the regulation of cooperation and aggression in the context of group living is the eusocial insects. Eusociality, which is typified by extreme cooperation including a reproductive division of labour whereby some individuals reproduce and others completely forgo reproduction to act as helpers, has evolved multiple times in the insects (Wilson 1971). For example, in highly eusocial honey bees, cooperation has reached extreme levels, with highly specialized castes including large, fecund queens, specialized workers, and a life cycle that requires colonies to be founded by a swarm of workers and a queen (Winston 1991). In such societies, aggressive behaviour is strongly directed towards non-colony members and intruders in colonies with fully functional queens (but see Visscher 1996), with relatively little conflict within colonies. In contrast, in the primitively eusocial *Polistes* paper wasps, cooperation and conflict within colonies are regularly displayed at different time points during the colony life cycle, i.e., at colony founding and in mature colonies (Reeve 1991). These features have made *Polistes* wasps a model system for the study of both conflict and cooperation, and their role in the evolution of eusociality (Jandt et al. 2014).

In the well-studied common European paper wasp, *Polistes dominula*, cooperation and aggression occur during multiple stages of colony development—during colony founding when multiple, fecund females may start a nest together, and later, after worker emergence, in the form of mother–daughter or sister–sister aggression (Reeve 1991). Interactions between co-foundresses in *Polistes* are especially easy to observe and are very well- documented; in fact, the first observations of dominance hierarchies in an invertebrate were made in *Polistes dominula* (Pardi 1948). During colony foundation, a single, mated female (foundress) may found a colony alone, constructing and defending the nest, foraging for food and nesting material, and laying eggs and caring for developing larvae. This strategy is referred to as single founding (Reeve 1991). Alternatively, multiple, often related females may team up and jointly found a colony, referred to as multiple founding. In such situations, one foundress is behaviourally more aggressive and dominant, and lays the majority of the eggs on the nest. The other foundresses are typically subordinate, with smaller ovaries and fewer eggs laid in general (but see Leadbeater et al. 2011), and spend more time foraging for food and tending brood (West-Eberhard 1969). In many species, such as the well-studied European *Polistes dominula*, there is variation within the species, with both single and multiple founding nests occurring in the same population. Cooperative colony founding may enhance the chances of colony survival, especially in challenging environments with high risk of predation or high intra-specific competition (Starks and Fefferman 2006).

Despite the broad knowledge on the behavioural patterns associated with colony founding behaviour in *P. dominula*, mechanisms of molecular regulation for the physiological and neural changes associated with cooperation and aggression are largely unknown. Initial studies showed that increased juvenile hormone (JH) and ecdysteroids are associated with reproductive dominance (Röseler et al. 1984), and increased ovary activation (Röseler et al. 1985). More recently, differences in brain neuroanatomy have been associated with caste and dominance status (O’Donnell and Bulova 2017). Other studies on North American *Polistes* wasps have characterized patterns of gene expression associated with reproductive dominance (Toth et al. 2014) and social recognition (Berens et al. 2016), but to date we are lacking information relating to the genetic basis of aggressive and cooperative behaviour in these wasps. In other social insects, there have been large transcriptomic studies that looked at global gene expression patterns associated with aggressive interactions during colony founding in fire ants (Manfredini et al. 2013), with dominance in the small carpenter bee (Withee and Rehan 2017), and with aggressive behaviour in honey bees in the context of colony defence (Alaux et al. 2009). Finally, studies on bumblebees have investigated the role of juvenile hormone (JH) and the gene *vitellogenin* in regulating intra-colony aggressive interactions among workers (Amsalem et al. 2014). In these studies, *vitellogenin* appears to be a good candidate for mediating social interactions among colony members, but it is challenging to clearly separate its function as a regulator of social behaviour from its role as a core reproductive gene (reviewed in Lockett et al. 2016). This has been achieved in a series of studies on honey bee workers, where the behavioural function of Vitellogenin is uncoupled from its reproductive role, as honey bee workers do not engage in reproductive activity. Here, Vitellogenin appears to regulate important behavioural functions, such as the onset of foraging behaviour, foraging specialization and lifespan (Nelson et al. 2007).

Our goal was to use a candidate gene approach, informed by prior studies on other social insects, to identify brain-expressed genes associated with cooperation and/or aggression in *P. dominula*. First, we used previously published data from *P. metricus* and fire ants to identify consistently expressed and potentially conserved genes associated with stable dominance status in insects—resulting in two lists of candidate genes, one for cooperation and one for aggression. Then we examined brain expression patterns of these candidate genes in individuals of *P. dominula* that were actively involved in dominance interactions to determine whether these genes are associated with different levels of aggressive behaviour. Finally, we used transcriptomic datasets from a previous study on *P. dominula* to provide a broader genomic/gene network based assessment of the interrelationships between key genes in the regulation of cooperation and aggression in this wasp.

## Materials and methods

### Selection of candidate genes for cooperation and aggression

In order to identify candidate genes related to cooperation and aggression in *P. dominula*, we proceeded by selecting genes from previous studies that showed consistent associations with reproductive dominance in wasps and ants. We focused on two previously published studies on the transcriptomic basis for nest founding behaviour in paper wasps and fire ants. These were the only studies available at the time when we designed the experiment that explored global changes of gene expression associated with dominance behaviour in foundresses of social insects (but see Helmkampf et al. 2016 for another more recent study on the transcriptomics of ant foundresses). The first study (study “A”, Toth et al. 2014) investigated the global patterns of gene expression associated with dominance hierarchies in *Polistes metricus*, a paper wasp closely related to *P. dominula*. This study analysed global gene expression both in the brain and in the ovaries of 5 groups of wasps: two groups during the colony founding phase, i.e. (1) dominant foundresses (DF) and (2) subordinate foundresses (SF), and three groups during the working phase of the colony, i.e. (3) reproductive functional queens (Q), (4) dominant workers (DW) and (5) subordinate workers (SW). The second study (study “B”, Manfredini et al. 2013), examined colony founding in the fire ant *Solenopsis invicta* and compared global expression of genes in whole bodies of different queen phenotypes. There were 3 groups of interest in this study: (1) queens from single foundress colonies, (2) winners, who were queens from pair-founding associations soon to become queens, and (3) losers, who were queens from pair-founding associations destined to succumb to winners.

For each study, we retrieved the list of unique genes that were differentially expressed in at least one comparison of interest: 498 genes for *P. metricus* (considering only the brain genes) and 4081 for *S. invicta* (Supp Tables 1, 2). We converted, where possible, the identifier for the wasp/ant genes into the *Drosophila melanogaster* best BLAST hit (“FB” IDs), using available gene annotations for these species: this produced 396 converted genes for *P. metricus*, and 2790 converted genes for *S. invicta*. With gene identifiers consistent across the two studies, we performed an overlap analysis to obtain a list of genes that were differentially expressed in both studies (Supp Table 3). We performed a Gene Ontology (GO) analysis on this set of shared genes to confirm that it included behaviourally relevant genes (see Supp Table1). We selected 34 genes that clearly showed differential expression in aggressive and cooperative phenotypes for both studies (Supp Table 4). The 34 genes were differentially expressed in: (a) DF and DW (aggressive) vs. SF and SW (cooperative) wasps AND (b) pair-founding associations (aggressive) vs. single founding (non-aggressive) ants, and/or winners (aggressive) vs. losers (cooperative). We further reduced the number of candidate genes to 12, by retaining only genes with relevant annotations on their putative functions in other organisms (Supp Table 5). This was the pool of genes that we used to identify the best candidates for cooperation and aggression, i.e. genes that were consistently up-regulated in cooperative phenotypes of paper wasps and fire ants, or genes that were consistently up-regulated in wasps and ants engaging in aggressive interactions (3 and 2 genes, respectively, see “Results” section “Candidate genes for cooperation and aggression” for full details and Table 1). We added a third gene for aggression related to octopamine signalling, as evidence became available while designing the experiment on the role of octopamine as a mediator of founding behaviour in ant queens (Koyama et al. 2015). Finally, we also investigated the expression patterns of 3 additional genes linked to the activity of *vitellogenin* and juvenile hormone (JH), because both Vitellogenin and JH are well known key players regulating behavioural and reproductive maturation in social insects (Amdam et al. 2003; Robinson and Vargo 1997). The functional link between Vitellogenin and JH in social insects was first hypothesised by Amdam and Omholt (2003), and the implications of this link at the behavioural level were documented in subsequent studies (Guidugli et al. 2005; Nelson et al. 2007).

### Primer design for candidate genes

We designed primers to perform quantitative real-time PCR (qPCR) on candidate genes for cooperation and aggression. We obtained the *P. metricus* sequences for the candidate genes from study A and we used them to retrieve the orthologue sequences in the *P. dominula* genome (Standage et al. 2016). For this purpose, we used the *P. dominula* genome deposited on the virtual genome annotation platform GDB (version PdomGDB r1.2). The reverse complement of the *P. dominula* genomic sequence was aligned to the *P. metricus* candidate gene sequence using the GDB: this step enabled us to select a portion for primer design that was overlapping, at least partially, with the region targeted by the microarray probe in study A. In this way, we made sure that we targeted with our qPCR approach the same exon(s) that coded for differentially expressed mRNAs in the microarray analysis. We used the web-tool Primer3plus (http://www.bioinformatics.nl/cgi-bin/primer3plus/primer3plus.cgi) to design forward and reverse primers for the targeted regions (Supp Table 6). We performed a BLAST screening of the designed primers to make sure that they did not target other regions in the *P. dominula* genome not associated with the genes of interest.

### Sampling of wasp colonies and lab maintenance

Incipient colonies of *P. dominula* were sampled in June 2015 at 4 sites: Ames IA town centre (42°01′33.2"N 93°36′55.4"W), Iowa State University Campus (42°01′36.5"N 93°38′55.7"W), University of Minnesota, Minneapolis Campus (44°58′21.1"N 93°13′49.0"W) and University of Minnesota, Saint-Paul Campus (44°59′09.7"N 93°10′53.6"W). Nests were spotted in the daytime and collected at night, to make sure that all the wasps were present on the nest at the time of collection. Nests and wasps were temporarily stored in plastic containers, provided with water and sugar and transported to the rearing facility as soon as possible. In total, we sampled 73 colonies with a single foundress and 10 multiple founding colonies, where the number of wasps on nest ranged from 2 to 5.

Colonies were housed in a rearing facility on ISU Campus. Nests were fixed to the roofs of large Plexiglas™ cages (28 × 28 × 28 cm) as described in (Daugherty et al. 2011) and wasp foundresses were paint marked for later identification. Rearing conditions were 12 h light/dark, 30 °C ± 2 and 50% humidity. Colonies were maintained in these conditions for 2 weeks. During this period, all pupae that were observed in multiple founding nests were removed to prevent the emergence of workers that could impact established social hierarchies among co-foundresses. Workers were allowed to emerge in single founding nests as their presence should not affect the social dominance status of the foundress.

### Classification of wasp phenotypes

Two weeks after installing wasp colonies in the rearing facility, all wasps present on all nests were flash frozen in liquid nitrogen. Wasps on single founding nests included the single foundress queen plus all workers emerged in the lab. All specimens were sampled between 10 am and 12 pm on the same day, and immediately stored in a − 80 °C freezer for later analyses. For multiple founding nests, we recorded the position of the wasps in the cage at the moment of collection to infer a first indication of their possible social rank (West-Eberhard 1969): on the nest = dominant wasp; foraging outside the nest = subordinate wasp. After collection, we performed a series of morphological and physiological measures to gather more detailed information on the social rank of co-foundresses by analysing their reproductive potential (Supp Table 7). First, we measured gaster size (width, length and the two combined) with an electronic caliper as a proxy for general size of the wasp: larger foundresses are more likely to have better fighting and reproductive abilities (Reeve 1991). Then, we dissected gasters and we quantified the amount of fat bodies covering the inner surface of the gaster cuticular layers. Fat bodies are essential for mated females to survive during the winter diapause and to facilitate spring emergence and colony founding: successful foundresses typically display large amounts of fat bodies in their gaster (Markiewicz and O’Donnell 2001). We ranked fat bodies according to a previously developed protocol (Beani et al. 2011): large = 1, intermediate = 2, poor = 3. Third, we isolated ovaries and we ranked their size (large, intermediate, small), and we recorded the presence of eggs and their development status according to the same protocol as above (one or more mature eggs = 3, immature eggs = 2, no eggs = 1). Both these measures are directly correlated to the reproductive potential of a wasp foundress (Bohm 1972). For each colony, we labelled as the dominant on nest the female that scored the highest for a combination of all morpho-physiological measures (see “Results” and Supp Table 8); the subordinate was designated as the individual that scored second.

### Brain dissections and RNA isolation

We isolated total RNA from individual wasp brains. Wasp heads were first freeze-dried for 60 min at 0.315 Torr on a 2.5 L Benchtop Labconco Freeze Dryer. Thereafter, heads were placed on a dissection plate on dry ice and we removed the outside cuticle with a fine scalpel. Exposed brains were separated from the head capsule, maintained in dry ice and immediately frozen at − 80 °C. To isolate total RNA we used the RNeasy Mini kit (Qiagen) and followed standard manufacturer instructions. We added a step of DNase treatment (QIAGEN) to remove possible contamination with genomic DNA. We checked quality and quantity of RNA samples with a spectrophotometer (NanoDrop 2000 and Qubit instruments). Only samples that passed quality check (260/280 ratio > 2) were selected for follow-up analyses. These samples were dried in a Labconco FreeZone 2.5L Lyophilizer, and resuspended in RNase-Free Water (QIAGEN) to a concentration of 40 ng/µl.

### Gene expression analyses

We started with equal amounts of total RNA from each sample to obtain cDNA with the SuperScript® III First-Strand Synthesis SuperMix (Invitrogen). We reverse transcribed 200 ng of RNA in a 20 µl volume reaction and we followed manufacturer instructions to setup the synthesis reaction. We diluted newly synthesized cDNA 4 times (final concentration = 2.5 ng/µl) and checked sample concentrations on the Nanodrop spectrophotometer to confirm that the synthesis reactions occurred with comparable efficiency. We added a non-enzyme control (NEC) to our set of reactions to exclude any possible amplification of aspecific material during the follow-up analyses.

We used 2 µl of cDNA to quantify gene expression levels on a CFX384 Touch™ Real-Time PCR Detection System (Bio-Rad) instrument following an established protocol validated in the Toth Lab (Berens et al. 2016). Briefly, we prepared 10 μl volume reactions with the 2X SYBR Green Master Mix (Applied Biosystem). Samples were run in triplicates (to control for technical errors) and across multiple plates: we added an interplate calibrator to each plate so that we could standardize the analysis of gene expression across multiple plates. We also added serial dilutions of genomic DNA from *P. dominula* to create standard curves at the following concentrations: 10,000, 1000, 100, 10 and 1 pg/µl. qPCR conditions were: 95 °C for 10 min, 40 cycles of 95 °C for 15 s followed by 65 °C for 1 min (for quantification), then gradual decrease from 65 °C to room temperature over 10 min (for melting peak analysis). We used two control genes that had been validated for *P. dominula* in a previous study: *actin* and *elongation factor 1* (Geffre et al. 2017).

### Statistical analyses

To analyse qPCR data, we obtained Cq values for each wasp phenotype per candidate gene and we averaged across the 3 technical replicates: outliers (i.e., individual replicates that had a Cq value of 0.5 higher or lower than the average) were manually removed beforehand. Averaged values were then normalized against the control genes *actin* and *elongation factor 1* (variance = 0.166 and 0.185, respectively). Normalized gene expression values (Supp Table 9) were analysed in SPSS Statistics (IBM, version 21). We performed a Multivariate Analysis of Variance (MANOVA) across all candidate genes to detect the effect of wasp phenotype on global gene expression, and then we performed LSD post hoc tests to detect significant differences between pairs of phenotypes for each gene that we tested. Finally, we also performed a Correlation analysis to identify patterns of co-expression among candidate genes. We corrected for multiple comparisons using Bonferroni (significance threshold = 0.005).

Hierarchical clustering analyses and heatmaps were obtained in R using the packages “gplots” and “RColorBrewer”: normalized gene expression data were scaled beforehand. Analyses of possible effects of fat body rank, ovary size, egg development and wasp location on gene expression were performed using General Linear Models (GLM) in SPSS. We built a model for each factor and added the measures of gaster size (length, width, length × width) as covariates in the design (Supp Table 10). We corrected for multiple comparisons using Bonferroni (significance threshold = 0.012).

To build the network of global gene expression in the *P. dominula* head we obtained expression levels from a head RNA-seq experiment described in Standage et al. (2016). We restricted the starting material to the 6298 genes that were queen-biased in that study, i.e. genes that showed more than onefold higher expression levels in queen vs. worker heads. We performed weighted gene co-expression network analysis (WGCNA) using a similar protocol as in Manfredini et al. (2017) with default settings and soft thresholding power = 30. We used VisANT (Hu et al. 2013), to visualize the network structure.

Gene Ontology (GO) analyses were performed with Blast2GO (version 4.1.9). Functional Annotation Clustering of GO terms resulting from the overlap between study A and study B was performed in DAVID (Huang et al. 2008) considering only “biological processes” and using the default *Drosophila* population background. GO terms associated with *vitellogenin*-correlated genes were visualized using REVIGO (Supek et al. 2011). We restricted this analysis to “biological processes” only and to GO terms obtained through the InterPRO database.

## Results

### Candidate genes for cooperation and aggression

Our comparison of previous studies identified a set of 52 genes with similar expression between paper wasps (brain tissue) and fire ants (whole bodies) undergoing the establishment of social hierarchies during colony founding (see Materials and methods). A functional analysis of the GO terms associated with these shared genes resulted in 31 clusters where at least one GO term was significantly enriched (*p* < 0.05). Many key biological functions were overrepresented in this set of GO terms, such as behaviour, neurogenesis, ageing, reproductive process and mating (see Supp Table 11). These findings provided support for isolating behaviourally relevant candidates for cooperation and aggression in *P. dominula* within this pool of shared genes.

We identified three top candidate genes for cooperation: *alpha-coatomer protein, Inositol-3-phosphate synthase* and *rasputin*. In the brain of *P. metricus*, these genes show up-regulation in subordinate foundresses of multi-queens associations and also in subordinate workers of established colonies (Toth et al. 2014): this suggests they might mediate cooperative behaviour in paper wasps. In *S. invicta*, the same genes consistently show down-regulation in both winners and losers during the peak of the conflict phase in pair-founding associations, when active fights between nest mate queens occur (Manfredini et al. 2013). This suggests these genes are active during the initial phase of cooperation among fire ant queens and they are turned off when open conflict starts (see Table 1 for details on expression patterns of these genes in previous studies). Available information for the 3 candidate genes for cooperation can be summarized as follows:

- *alpha-coatomer protein* (*alphaCop*) is a regulator of lipid homeostasis in *Drosophila* that appears to be evolutionary conserved across different organisms (Beller et al. 2008);
- *inositol-3-phosphate synthase* (*Inos*) is a gene involved in the regulation of circadian rhythm in *Drosophila* and also associated with behavioural maturation in honey bees (Fu and Whitfield 2012);
- *rasputin* is a gene coding for an evolutionarily conserved RNA-binding protein believed to function as a link between Ras signalling and RNA metabolism; this gene participates in the process of oogenesis in *Drosophila* (Costa et al. 2013).

**Table 1:**
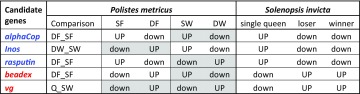
Selection of best candidate genes for cooperation (blue) and aggression (red) in *P. dominula* based on previous studies

Candidate genes for cooperation: up-regulated (UP) in *P. metricus* subordinate foundresses (SF) and subordinate workers (SW), and down-regulated (down) in *S. invicta* pair-founding queens (winners and losers). Candidate genes for aggression: up-regulated in *P. metricus* dominant foundresses (DF) and dominant workers (DW) and up-regulated also in *S. invicta* pair-founding queens. Grey cells indicate comparisons that were not statistically different in the study of interest. “Comparison” indicate the significant pairwise comparison for the gene of interest in the *P. metricus* study: Q = single foundress

For the aggression component, we identified two top candidate genes suitable for follow-up analyses in *P. dominula: beadex* and *vitellogenin*. These genes are up-regulated in dominant foundresses and dominant workers of *P. metricus* (Toth et al. 2014), suggesting that they might mediate the aggressive behaviour that these wasps perform to maintain their social status. The same genes are also up-regulated in winner and loser foundresses of *S. invicta* (compared to single foundresses) at the peak of the conflict phase (Manfredini et al. 2013), indicating that they might play an important role in the regulation of the aggressive interactions among fire ant queens (see Table 1 for details on expression patterns of these genes in previous studies). The third candidate gene for aggression was selected based on recent evidence from the literature (see below). Available information for the 3 candidate genes for aggression can be summarized as follows:

- *beadex* is a modulator of acute cocaine sensitivity and circadian locomotor rhythmicity in *Drosophila* (Tsai et al. 2004);
- *vitellogenin (vg)* is a highly conserved gene in the animal kingdom that codes for a precursor protein of egg yolk; this gene is particularly important in social insects as in addition to its function in reproduction it has been co-opted for other functions like behavioural maturation and worker division of labour (Amdam et al. 2003);
- *octopamine receptor beta-2* (*Octbeta2R*) is the receptor of octopamine (OA), a well conserved biogenic amine that in insects regulates olfactory and visual systems, motor system and reproductive system, and has been linked to aggressive behaviour; a recent study shows that OA promotes the disappearance of cooperation in founding queens of the ant *Polyrhachis moesta* (Koyama et al. 2015).

As *vg* appeared to be the gene with expression patterns that best matched dominance hierarchies in our wasp samples, we also investigated additional genes linked to *vg*, and to the major insect endocrine regulator juvenile hormone (JH). This decision was also motivated by the fact that prior studies on *Polistes* and other social insects suggests *vg* (Toth et al. 2014) and JH (Röseler et al. 1984) are related to reproductive and social dominance. The additional genes are:

- *vitellogenin receptor* (*vgr*), mediating the uptake into the developing oocytes of the newly synthesized Vg (Lu et al. 2009);
- *juvenile hormone esterase* (*jhe*), a key component in the metabolism of JH (Mackert et al. 2008);
- *juvenile hormone acid O-methyltransferase* (*jhamt*), a key enzyme in the biosynthesis of JH (Shinoda and Itoyama 2003).

### Classification of wasp phenotypes

We obtained one dominant and one subordinate foundress from 7 multiple founding associations out of the 10 that we collected. Dominant foundresses were the wasps with the highest morpho-physiological score within each association (median score = 15, Supp Table 10): they were on the nest at the time of collection, had large ovaries with mature eggs (i.e. they were the principal egg-layer on the nest), abundant fat bodies and the largest gaster (Table 2). Subordinate foundresses instead (median score = 11) were off the nest at the time of collection, they engaged in foraging and they had small to intermediate ovaries and small gasters. Patterns of egg development were mixed, but 6 out of 7 subordinates showed signs of reproductive activation, i.e., eggs ranked 2 or 3 (see Table 2). Only in one instance was there clear disagreement among measures: in colony F, the two foundresses had very similar scores (15 and 16) and we labelled as dominant the wasp on the nest, despite the fact that she had a smaller gaster than her nestmate. This is not unexpected as size is not a perfect predictor of dominance status in *Polistes* (Sullivan and Strassmann 1984).

**Table 2:**
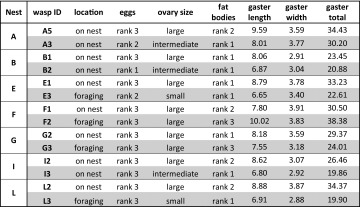
Full characterization of 14 foundresses from 7 multiple founding nests that were used for the analysis of brain expression of candidate genes

Unshaded cells correspond to wasps that were assigned to the dominant rank, while shaded cells correspond to wasps that were labelled as subordinate

For the 7 single founding colonies, we obtained 1 queen (individual wasps present on the nest at the time of colony collection) and 1 worker (wasps emerged in the lab after collection) per colony. These workers were no more than 10 days old.

### Patterns of brain gene expression in relation to behaviour and physiology

**Fig. 1:**
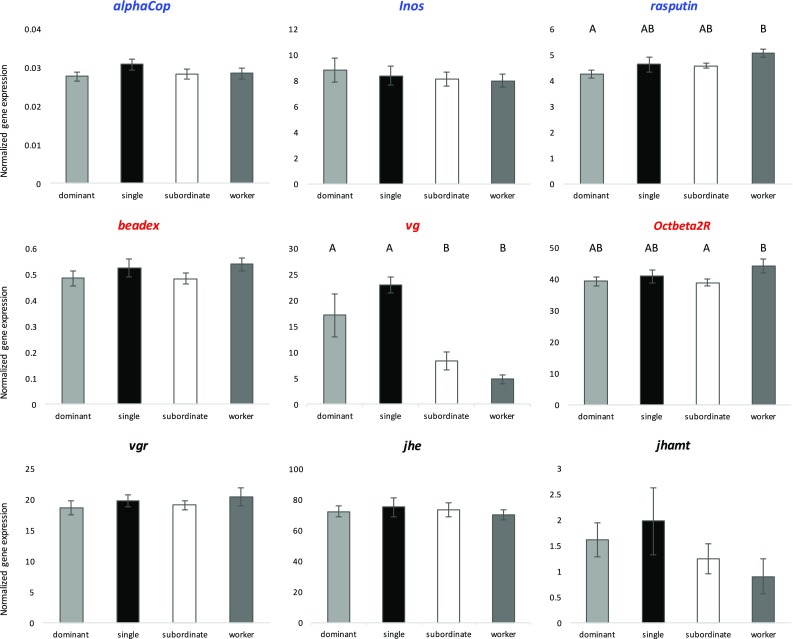
Brain gene expression in *P. dominula* females (dominant foundresses, single foundresses, subordinate foundresses and workers) for 9 different candidate genes, as determined by qPCR, and normalized to control genes *actin* and *elongation factor 1*. Candidate genes for cooperation are in blue, candidates for aggression are in red and the additional *vg*/JH-related genes are in black. Means +/− standard errors are reported. Different letters on top of bars correspond to statistically significant differences between two wasp phenotypes (*p* < 0.05, MANOVA with LSD post hoc tests)

Among the candidate genes for cooperation, only *rasputin* appeared to be affected by wasp rank (MANOVA *p* = 0.055). *Rasputin* expression was significantly higher in workers compared to dominant foundresses (MANOVA with LSD test, *p* = 0.007), but it was not significantly different for any of the other pairwise comparisons (MANOVA with LSD test, *p* > 0.05). Among the candidate genes for aggression, *vg* was strongly related to wasp rank (MANOVA *p* < 0.001): this gene had its highest expression in single foundresses, followed by dominant foundresses, then subordinate foundresses, and lastly, workers (Fig. 1). Both single and dominant foundresses had significant higher *vg* levels compared to workers (MANOVA with LSD test, *p* < 0.001 and *p* = 0.001, respectively) and subordinate foundresses (MANOVA with LSD test, *p* < 0.001 and *p* = 0.016, respectively). Another candidate gene for aggression, *Octbeta2R*, had significantly higher expression in workers compared to subordinate foundresses (MANOVA with LSD test, *p* = 0.048). For all the other genes, we did not detect any significant difference across wasp phenotypes (Fig. 1).

**Fig. 2:**
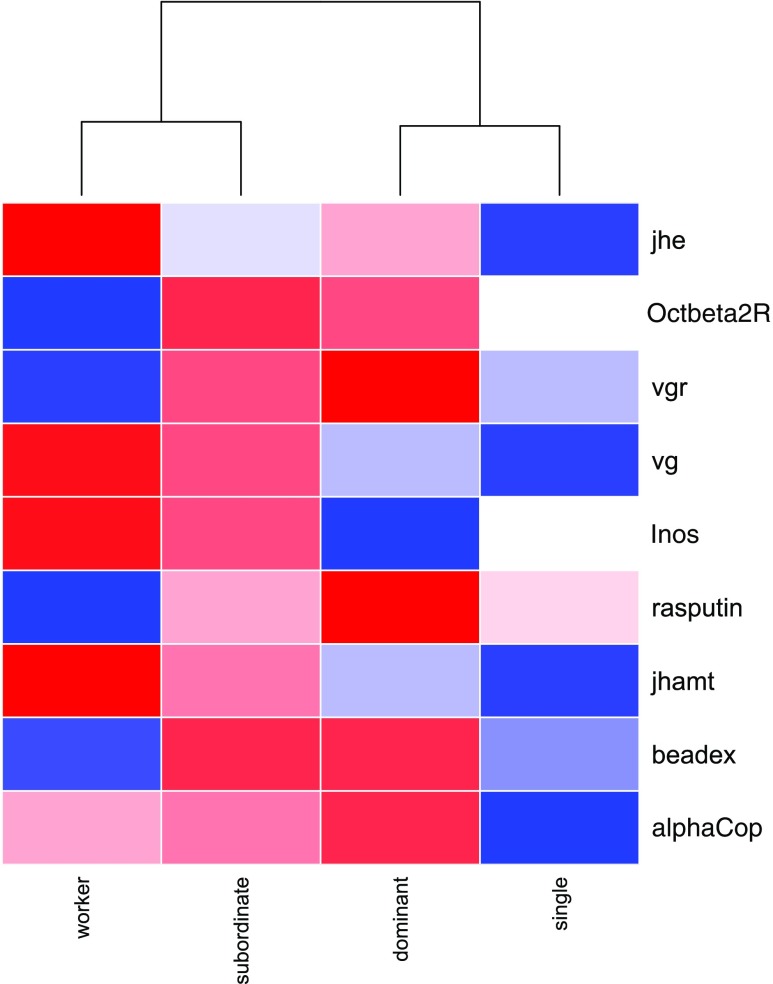
Clustering analysis of the four wasp phenotypes according to patterns of brain gene expression. The heatmap is colour-coded based on average levels of expression of each candidate gene: red = down-regulated; blue = up-regulated

Clustering based on group mean expression (of all genes) revealed that dominant foundresses clustered with single foundresses while subordinate foundresses clustered with workers (Fig. 2). This confirms that social rank is an important factor that is strongly linked to patterns of expression of candidate genes in the brain, while social environment (or colony of origin) was not related to expression of these genes. Clustering candidate genes based on their pattern of expression produced two main clusters: one with *jhe, Inos, alphaCop, beadex, rasputin* and *jhamt*, and a second, smaller cluster with *vg*, *vgr* and *Octbeta2R* (Supp Fig. 1). In addition, we calculated correlation values for pairs of genes to identify genes that shared the most similar expression patterns. Several positive correlations were detected, and those that survived Bonferroni correction for multiple comparisons (*p* < 0.005) were the following: *beadex*/*rasputin* (Pearson = 0.64), *beadex*/*vgr* (Pearson = 0.68), *beadex*/*jhe* (Pearson = 0.54), *Octbeta2R*/*rasputin* (Pearson = 0.86), *Octbeta2R*/*vgr* (Pearson = 0.72), *rasputin*/*vgr* (Pearson = 0.73), and *vgr*/*jhe* (Pearson = 0.60, Table 3).

**Table 3:**
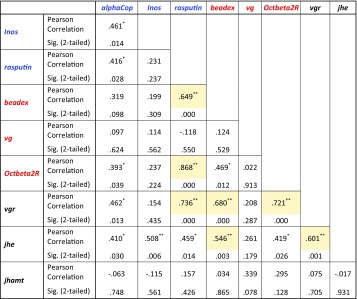
Analysis of correlation of expression for candidate genes

Candidate genes for cooperation are in blue, candidates for aggression are in red and the additional *vg*/JH-related genes are in black. Correlations that are significant after Bonferroni correction for multiple comparisons are highlighted in yellow (significance threshold = 0.005)

We also explored whether any other individual wasp measure (wasp location in the cage, fat body rank, ovary size and egg development) was associated with patterns of gene expression in the brain. We detected a positive association between fat body score and expression of *jhamt* (GLM *F* = 4.512, *df* = 5, *p* = 0.03). We also found significant positive correlations between measures of gaster size (including gaster length, gaster width, and length × width) and *beadex* expression (GLM, *p* < 0.05). However, none of these associations survived Bonferroni correction for multiple comparisons (*p* = 0.012). No other effects on brain gene expression were detected for ovary size, egg development or wasp location in the cage (data not shown).

### Localization and genomic context of *vg* expression in *Polistes dominula*

Our gene expression analyses (above) resulted in the identification of the brain gene coding for the yolk protein Vg as a strong correlate of aggression and social dominance in *Polistes* and this prompted us to further investigate the functional role of Vg in *Polistes* behaviour. First, we examined additional data to better understand the possibility of *vg* mRNA expression in the brain (vs other parts of the head). Second, we used previously published RNA-seq data to understand *vg* mRNA expression in a genomic context within gene networks.

Although Vg is a deeply conserved egg yolk protein (Tufail et al. 2014), there have been several recent reports showing *vg* mRNA can be expressed in head tissues in insects (Amsalem et al. 2014; Gospocic et al. 2017; Lockett et al. 2016; Münch et al. 2015; Roy-Zokan et al. 2015), and the presence of Vg protein and mRNA expression of the receptor *vgr* within glial cells in the brain. These data suggest Vg signalling within the head and within the brain has the potential to mediate suites of physiological and behavioural traits related to reproduction, maternal care, and sociality (Roy-Zokan et al. 2015). We examined *vg* mRNA levels in both brains and heads of individual wasps from a different set of 20 single foundresses that were processed as described in the “Materials and methods” section above. Expression levels of *vg* were 1.14 times higher in the head tissue than the brain, based on relative quantification with respect to control genes (Supp Fig. 2).

**Fig. 3:**
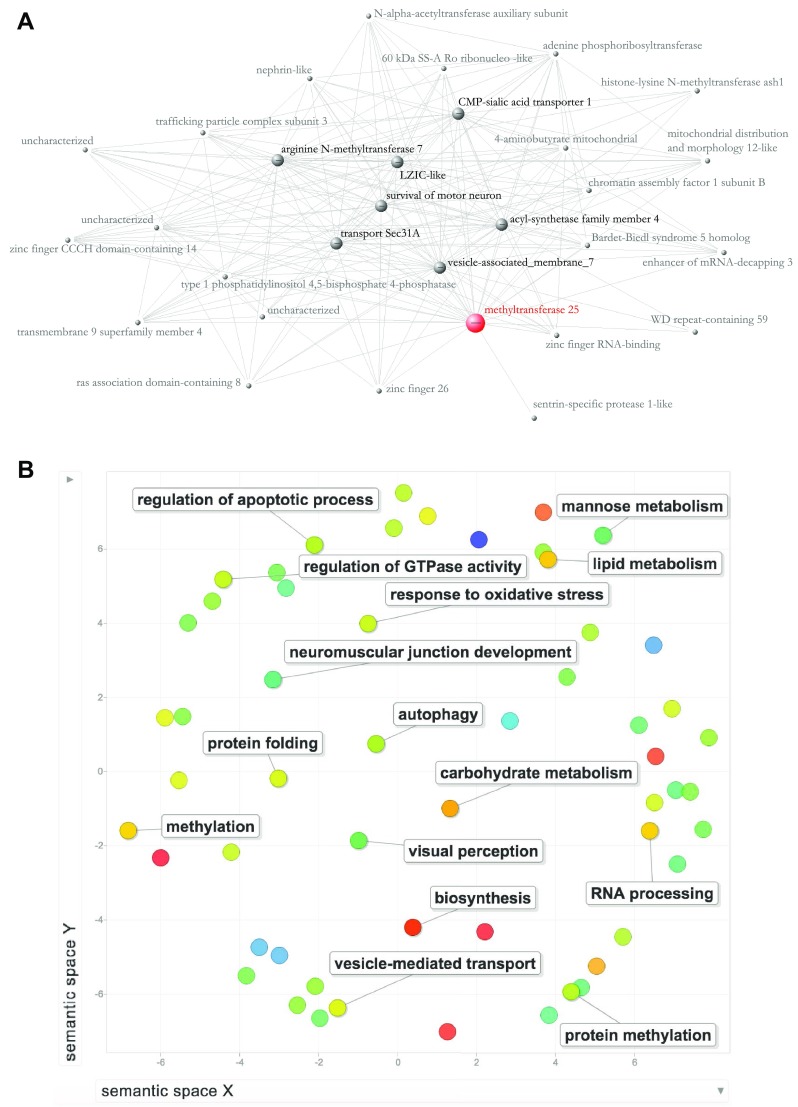
**a **Visualization of the 30 most connected genes in the wasp head network – obtained using the module of 6283 queen-biased genes from the WGCNA analysis on the Standage et al. dataset (2016). The genes visualized in the network correspond also to the genes that have the highest correlation with *vg* expression levels in the *P. dominula* head. Red node = major hub (most connected gene); large grey nodes = secondary hubs. **b** REVIGO analysis of GO terms associated with the 1999 *vg*-correlated genes in the wasp head. Output limited to Biological Processes only. The most meaningful terms for each cluster are reported in the figure—while for a full list of GO terms in each cluster please see Supp Table 13. Node colours are coded according to the size of the respective GO terms in the Gene Ontology database: warmer colours indicate larger terms

To better understand the genomic context of *vg* expression in *P. dominula*, we performed *vg*-centred exploratory analysis of gene co-expression networks based on previously published RNA-seq data from queen and worker *P. dominula* heads (Standage et al. 2016). The WGCNA approach produced two clusters of genes, each cluster containing genes that shared similar patterns of expression: a first large cluster encompassing most of the genes that were part of the analysis (6283 genes including *vg*) and a much smaller cluster of only 15 genes. Within the large cluster, 1999 genes were correlated with *vg* with correlation values ranging from 0.429 to 0.595 (see Supp Table 12, lower cut-off dictated by the soft thresholding power that we chose, see “Materials and methods”). A Gene Ontology analysis of *vg*-correlated genes identified 523 unique functional GO terms associated with these genes. Among the GO terms in the category “biological processes” some are of particular interest e.g., protein methylation, stress response, and metabolism of lipids and carbohydrates (Fig. 3B and Supp Table 13). In addition, we visualized the network of the 30 most connected genes within the same cluster (Fig. 3a): these are also the genes showing the highest correlation to *vg* (Supp Table 12). This group of genes include the major hub (most connected gene) *methyltransferase-like protein 25*, which catalyzes methylation reactions [not specific to DNA methylation, which is very low in *P. dominula* (Standage et al. 2016)]. We also identified 7 minor hub genes: *LZIC-like*, a gene involved in neuronal development (Clements and Kimelman 2005), *arginine N-methyltransferase 7*, an homolog of vertebrate PRMT7 responsible for histone methylation, *CMP-sialic acid transporter 1* (Eckhardt et al. 1996), related to nucleotide sugar transport in the Golgi apparatus, *vesicle-associated membrane 7* (Takáts et al. 2013), involved in autophagy and with roles in central nervous systems function, *transport Sec31A*, active in the endoplasmic reticulum–Golgi compartment of the cell (Tang et al. 2000), and *acyl-synthetase family member 4*, an enzyme involved in synthesis of fatty acids.

## Discussion

### Patterns of gene expression associated with cooperation and aggression

In this study, we provide new information about possible mechanisms of regulation of cooperation and aggression in *P. dominula* wasps, a model system for understanding sociality and its evolution. Overall, our combined data from all candidate genes suggest that patterns of expression of candidate genes depend more on social rank than on reproductive activation or social environment (Fig. 2). Dominant foundresses’ gene expression clustered together with single foundresses (which share high social rank, as both are at the top of the social hierarchy within their colony). Interestingly, we did not see a major effect of reproductive activation on gene expression (which would group single and paired-foundresses together) nor shared social environment (which would group dominant with subordinate foundresses on one side, and single foundresses with workers on the other side). This result stands in contrast to other studies on social insects in which the social environment had a massive effect on patterns of global gene expression (Malka et al. 2014; Manfredini et al. 2014; Wang et al. 2008). This suggests the collection of candidate genes we studied had some value as indicators of social rank.

From among the collection of genes that we analysed, we identified three top candidate genes with differential expression between *P. dominula* females that differed in dominance rank: *rasputin, Octbeta2R* and *vg*. Expression of *rasputin (rin)*, was highest in cooperative workers and lowest in dominant foundresses. However, expression levels in single and subordinate foundresses were intermediate, thus there was no simple relationship with wasp cooperative behaviour for this gene. The known functions of *rin* in model organisms suggest a potential role in behavioural regulation. *Rin* has roles in the regulation of reproduction and neural plasticity in adult *Drosophila* flies (Ivshina et al. 2014). *Rin* positively regulates Orb, a mRNA binding protein that is involved in long-term memory formation (Pai et al. 2013). *Rin*’s role in neural development in social wasps deserves further investigation in the future, including the possibility that it could affect brain plasticity and learning and memory in the context of cooperative behaviour. The second gene, *Octbeta2R*, is a receptor of octopamine (OA), a biogenic amine that has been linked to cooperative behaviour and aggression in ants (Koyama et al. 2015). Our results indicate that the expression levels of *Octbeta2R* mirror the levels of circulating OA as observed in the ant study: *Octbeta2R* was higher in members of an established colony (single foundresses and workers in our study) than in foundresses (although only the worker/subordinate comparison was significantly different in our study). As suggested in Koyama et al. (2015) for OA, low levels of *Octbeta2R* in foundresses might be related to acceptance of another foundress within a multi-foundress association. Higher levels of *Octbeta2R* in workers (reported in our study) could reflect the extreme aggressive behaviour that workers display when defending the colony against intruders. The yolk protein gene *vg* clearly showed the strongest correlation with rank and aggression in *P. dominula*, and we discuss these findings in detail below.

Although none of the other genes differed significantly in their expression levels across wasps of different social ranks, the expression pattern of one additional gene, *jhamt*, suggests a possible role in a rank-related phenotype. *jhamt* is involved in the synthesis of JH, and was numerically (though not statistically) highest in single foundresses, followed by the dominants, subordinates and workers. Interestingly, *jhamt* was also significantly correlated with larger abdominal fat bodies. Thus, *jhamt* may reflect high levels of JH in the haemolymph and could mediate increased fat storage, in line with past evidence that JH levels can be an indicator of “high-quality” in queen *Polistes* (Tibbetts and Izzo 2009).

### A potential role for *vitellogenin* in the regulation of aggression in social wasps

The patterns of expression of *vg* that we report in this study clearly mirror the social structure of *Polistes* wasps: *vg* is expressed at the highest levels in single foundresses, followed by dominant foundresses, subordinates and workers. Together with previous findings from *P. metricus* in stable dominance associations (Toth et al. 2014), as well as data from reproductively dominant fire ant queens (Manfredini et al. 2013), our data suggest *vg* could be a major regulator not only of reproductive physiology, as is well known, but also in brain regulation of aggressive, dominance behaviour during colony founding in social insects. Importantly, subordinate foundresses are reproductively active—even though at a lower level than dominants. The fact that this group has *vg* expression levels that are comparable to sterile workers (and not to reproductive dominant or single foundresses) is a strong indication that *vg* is correlated with aggressive behaviour, not just reproduction. These observations are in line with findings of Amsalem et al. (2014), in which *vg* expression levels were found to be highest in queen-less bumblebee workers that display aggressive behaviour towards nestmates, even before they activate their ovaries and start laying eggs. Nonetheless, our data are only correlative, so future studies should aim to manipulate *vg* levels in vivo and examine effects on dominance behaviour to establish whether there is a causal association. A similar approach has been performed in honey bee workers, where *vg* has been successfully knocked down via RNA interference through abdominal injection: this resulted in a significant modification of workers foraging onset and foraging preference (Nelson et al. 2007).

Although Vg was first described and is well known to be an egg yolk protein synthesized at high levels in the fat body and present in the ovaries of actively reproductive female insects (reviewed in Tufail et al. 2014), studies in honey bees suggest “non-traditional” modes of action and expression localization patterns. For example, Amdam et al. (2003) showed that honey bee workers can transfer Vg via royal jelly to brood, workers and queens, thanks to receptors for Vg localized in their hypopharyngeal glands. These findings have led the authors to suggest that *vg* has been co-opted for novel, social functions in highly eusocial species (Amdam et al. 2003). More recently, using in situ hybridization, Vg protein localization and *vgr* mRNA expression were detected within the brain of individual honey bees (Münch et al. 2015). Other studies have also suggested *vg* expression in the brains of other insect species such as burying beetles, and suggested this gene could regulate reproductive and socio-parental behaviours more broadly in insects (Roy-Zokan et al. 2015). The fact that we found very high expression of *vg* in non-brain tissues in *P. dominula* heads suggests the presence of *vg* transcripts in our samples may have been the result of high levels of *vg* in surrounding tissues. Thus, *vg* expression in the head, not the brain, could account for the patterns we observed. We did detect substantial levels of *vgr* in wasp brains (Fig. 1), leaving open the possibility that *vg* produced in fat body (or other head tissues) could be translated into Vg protein and then transported into the brain, binding to *vgr* and possibly regulating behaviour, as has been proposed for honey bees (Münch et al. 2015). Further work (such as *in situ* hybridizations) will be necessary to better understand localization of *vg* and *vgr* transcripts and their associated proteins in *Polistes*. The strong relationship between *vg* and dominance behaviour in this study suggests a possible role for head to brain Vg signalling in mediating social aggression in *Polistes* wasps.

### *vitellogenin* expression in a genomic and evolutionary context

Although in this study *vg* is strongly associated with aggressive behaviour, it is likely acting in concert with differential expression of many other genes. Our gene co-expression network analysis suggests *vg* expression is highly correlated with the expression of hundreds of other genes in the head, including many genes with functions in lipid and carbohydrate metabolism, protein methylation, and stress response. Lipid metabolism and stress response genes are also strongly correlated with caste differences in the head and the brain of *Polistes* (Standage et al. 2016; and Toth et al. 2014, respectively), thus there may be some common molecular determinants of dominance status and caste. In addition, the genes with the strongest correlation to *vg* in *Polistes* heads include two genes with neural functions, *LZIC-like* and *vesicle-associated membrane 7*. This suggests there may be some feedback between *vg* expression in the head and expression of genes controlling behaviour in the brain. The potential signalling function of *vg* in the context of behaviour has also been suggested in other insects (Lockett et al. 2016). Perhaps in *Polistes*, Vg could serve as an indicator molecule in the head that can signal reproductive status to the brain, resulting in downstream changes in brain gene expression that can regulate the frequency of adaptive behaviours, including aggression. We suggest this intriguing idea deserves further study in the future in *Polistes* and in insects in general.

The fact that *vg* was a strong predictor of social rank from our initial list of wasp-ant shared candidate genes, and also turned out to be the most robust gene expression associated with aggression in the current study, shows a strong and consistent association between *vg* and dominance in multiple insect species. Our finding that *vg* expression and/or signalling may be related to the regulation of aggressive behaviour in wasps also provides support for the “ovarian ground plan hypothesis” (West-Eberhard 1969), and the related “reproductive ground plan hypothesis” (Amdam et al. 2004). Under these ideas, mechanisms that regulate reproductive physiology/behaviour and maternal foraging/provisioning behaviour may be uncoupled during evolution, leading to the occurrence of queen-like reproductive and worker-like foragers. We suggest that in the evolution of sociality, Vg may have been co-opted not only for the regulation of worker behaviour in honey bees (Amdam et al. 2004), but also for the regulation of aggression and dominance in ants and wasps. In light of other recent studies suggesting Vg may have also been co-opted to regulate parental behaviour in beetles (Roy-Zokan et al. 2015), this study adds to the growing weight of evidence that Vg signalling in the head and brain may play a role in the regulation of deeply conserved insect behaviours. Thus, Vg is much more than a yolk protein, with properties of a signalling protein that may help to coordinate suites of organismal traits such as reproductive physiology and aggressive behaviour.

## Electronic supplementary material

Below is the link to the electronic supplementary material.

Supplementary material 1 (DOCX 1642 KB)

Supplementary material 2 (XLSX 593 KB)

## Gene names

*alphaCop*: *Alpha-coatomer protein*
*Inos*: *Inositol-3-phosphate synthase*
*jhamt*: *Juvenile hormone acid O-methyltransferase*
*jhe*: *Juvenile hormone esterase*
*Octbeta2R*: *Octopamine receptor beta-2*
*rin*: *Rasputin*
*vg*: *Vitellogenin*
*vgr*: *Vitellogenin receptor*

## Others

cDNA: Complementary DNA
Cq: Quantification cycle
GO: Gene ontology
JH: Juvenile hormone
mRNA: Messanger RNA
OA: Octopamine
*P. dominula*: *Polistes dominula*
*P. metricus*: *Polistes metricus*
qPCR: Quantitative real-time PCR
*S. invicta*: *Solenopsis invicta*
WGCNA: Weighed gene co-expression network analysis
