# Supplementary material for: Candidate genes for cooperation and aggression in the social wasp Polistes dominula
Source: J Comp Physiol A Neuroethol Sens Neural Behav Physiol. 2018 Feb 27;204(5):449–63. doi: 10.1007/s00359-018-1252-6 (PMC5907630; doi:10.1007/s00359-018-1252-6)
Supplement: Supplementary file 1 — Supplementary material 1 (DOCX 1642 KB) [file 359_2018_1252_MOESM1_ESM.docx]

Supplementary Information

**TABLES**

All supplementary tables are provided as an Excel file. Table contents and legends below.

**Table S1.** List of differentially expressed genes from Toth et al. 2014, comparing brain gene expression using microarrays between dominant and subordinate foundresses, solitary foundresses, queens, and workers of *Polistes metricus*.

**Table S2.** List of differentially expressed genes from Manfredini et al. 2013, comparing gene expression using microarrays between winners and losers in associations of pair-founding queens or among solitary foundresses of the fire ant *Solenopsis invicta*.

**Table S3.** List of genes that were differentially expressed in association with dominance and aggression in both *Polistes metricus* (Toth et al. 2014) and *Solenopsis invicta* (Manfredini et al. 2013).

**Table S4.** Genes related to dominance and aggression in both *Polistes metricus* (Toth et al. 2014) and *Solenopsis invicta* (Manfredini et al. 2013) that showed consistent patterns of expression in the two studies.

**Table S5.** Information on putative annotations, related Gene Ontology (GO) terms, and associated references for candidate genes derived from the overlap between the wasp and ant studies (Toth et al. 2014, Manfredini et al. 2013). Top 5 candidate genes for this study, as determined from a follow-up analysis, are highlighted.

**Table S6.** Primer sequences used for real time qPCR in this study, including 9 target genes and two internal control (“housekeeping”) genes.

**Table S7.**  Complete information for all wasps collected from multiple founding nests in this study, including position on nest, ovary size, fat bodies, gaster measurements, place of collection.

**Table S8.** Scoring table for wasp traits, and scores given to all multiple founding wasps included in this study.

**Table S9.** Raw data from real time qPCR analysis for all sampled individual wasps included in this study.

**Table S10.** Raw data used to examine correlations between gene expression and physiological and morphological features of individual wasps.

**Table S11.** List of GO terms associated with genes that were commonly differentially expressed between wasps and ants (Toth et al. 2014, Manfredini et al. 2013).

**Table S12**. List of 1999 genes that showed a significant correlation with *vg* expression in the *Polistes dominula* head, from Standage et al. 2016.

**Table S13.** REVIGO analysis of the 523 unique GO terms associated with the genes correlated with *vg* in the wasp head.

**FIGURES**

**Supplementary Figure 1.** Clustering analysis of the 28 wasps used in this study according to patterns of brain gene expression. The heatmap is colour-coded based on average levels of expression of each candidate gene: red = down-regulated; blue = up-regulated. dom = dominant foundress; mon = single foundress; sub = subordinate foundress; wor = worker.

**Supplementary Figure 2.** Levels of expression of *vg* brain vs. *vg* head. Indicated on the Y axis is the relative quantification of *vg* for brain and head tissues with respect to the control genes *actin* and *elongation factor 1*.

**
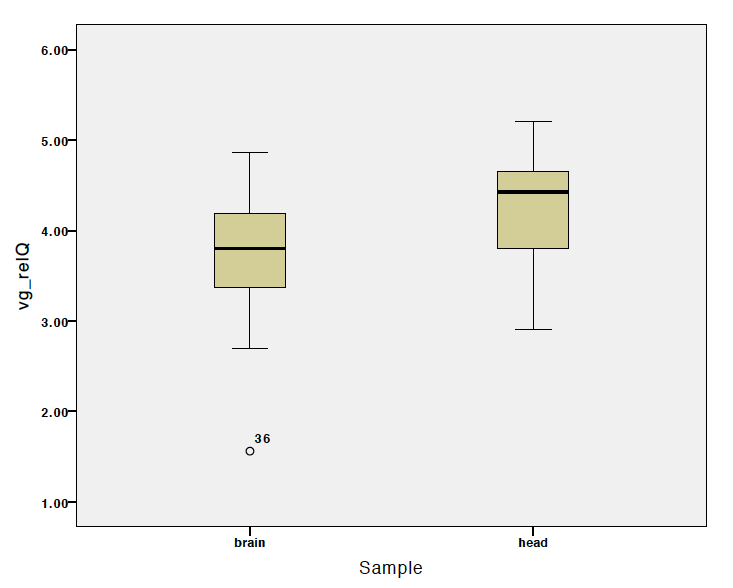
**
